# Impact of minocycline on outcomes of EGFR-mutant non-small cell lung cancer patients treated with EGFR-TKIs

**DOI:** 10.1038/s41598-023-35519-4

**Published:** 2023-05-23

**Authors:** Mari Tone, Kota Iwahori, Takayuki Shiroyama, Shinji Futami, Yujiro Naito, Kiyoharu Fukushima, Kotaro Miyake, Shohei Koyama, Haruhiko Hirata, Izumi Nagatomo, Hisashi Wada, Yoshito Takeda, Atsushi Kumanogoh

**Affiliations:** 1 Department of Respiratory Medicine and Clinical Immunology, Osaka University Graduate School of Medicine, Osaka, Japan; 2 Department of Clinical Research in Tumor Immunology, Osaka University Graduate School of Medicine, Osaka, Japan; 3 Department of Immunopathology, World Premier International Research Center Initiative (WPI), Immunology Frontier Research Center (IFReC), Osaka University, Osaka, Japan; 4 Integrated Frontier Research for Medical Science Division, Institute for Open and Transdisciplinary Research Initiatives (OTRI), Osaka University, Osaka, Japan; 5 Center for Infectious Diseases for Education and Research (CiDER), Osaka University, Osaka, Japan; 6 Japan Agency for Medical Research and Development – Core Research for Evolutional Science and Technology (AMED–CREST), Osaka University, Osaka, Japan; 7 Center for Advanced Modalities and DDS (CAMaD), Osaka University, Osaka, Japan

**Keywords:** Targeted therapies, Non-small-cell lung cancer

## Abstract

Minocycline is often administered prophylactically or therapeutically to non-small cell lung cancer (NSCLC) patients receiving epidermal growth factor receptor-tyrosine kinase inhibitors (EGFR-TKIs) for skin rash as an adverse event. We examined the effects of minocycline on the outcomes of EGFR-mutant NSCLC treated with first-line EGFR-TKIs based on a single-center retrospective analysis. In this retrospective cohort study, data were collected on NSCLC patients treated with first-line EGFR-TKIs between January 2010 and June 2021. The treatment efficacy of first-line EGFR-TKIs was compared between patients who received minocycline and those who did not. Median progression-free survival (PFS) with first-line EGFR-TKIs was significantly longer in the minocycline group (N = 32) than in the control group (N = 106); 714 (95% confidence interval CI 411–1247) days vs. 420 (95% CI 343–626) days, p = 0.019. A multivariate analysis including skin rash as a variable confirmed that the administration of minocycline for 30 days or longer correlated with good PFS and overall survival (OS) with first-line EGFR-TKIs (HR 0.44 [95% CI 0.27–0.73], p = 0.0014 and HR 0.50 [95% CI 0.27–0.92], p = 0.027, respectively). The administration of minocycline influenced good treatment efficacy with first-line EGFR-TKIs independently of skin rash.

## Introduction

Since the discovery of epidermal growth factor receptor (EGFR) mutations in lung cancer patients, the development of EGFR tyrosine kinase inhibitors (EGFR-TKIs) has increased the survival of patients with EGFR-mutant non-small cell lung cancer (NSCLC). After the development of first-line EGFR-TKIs (gefitinib and erlotinib)^1–4^ and second-line EGFR-TKI (afatinib and dacomitinib)^5–8^, a third-line EGFR-TKI (osimertinib) received FDA and EMA approval^9–11^. Although EGFR-TKIs show good efficacy against EGFR-mutant NSCLC, they are associated with unique adverse events. One of the common adverse events of EGFR-TKIs is skin rash. Due to its higher selectivity to the mutated receptor, osimertinib is associated with less severe skin toxicity than first- or second-line EGFR-TKIs^9,12^. To prevent or attenuate skin rash induced by EGFR-TKIs, minocycline is often administrated during EGFR-TKI treatments^13–15^. EGFR TKI-related skin rash in NSCLC patients correlates with a better treatment outcome than the absence of any grade of skin rash^16–20^. However, the effects of minocycline on the outcomes of EGFR-mutant NSCLC patients treated with EGFR-TKIs remain unclear. A recent retrospective nationwide registry study in Finland indicated that tetracyclines increased the survival of NSCLC patients treated with EGFR-TKIs^21^. Since this study was based on drug purchases in a prescription database, there were uncertainties regarding clinical data. Therefore, we herein examined the effects of minocycline on the outcomes of EGFR-mutant NSCLC treated with first-line EGFR-TKIs based on a single-center retrospective analysis.

## Results

### Baseline patient characteristics

**Figure 1:**
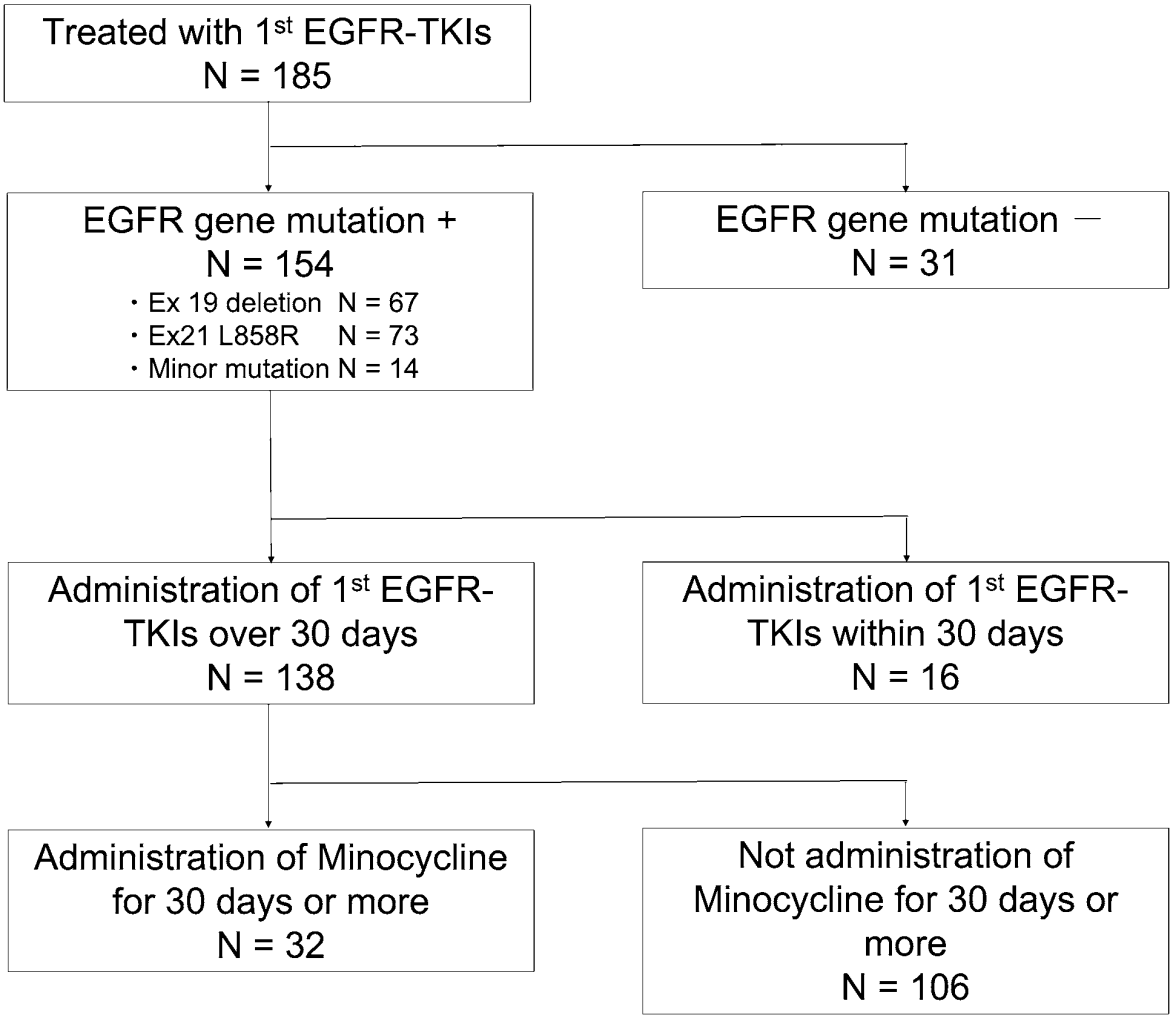
Characteristics of patients enrolled in the present study. *EGFR* epidermal growth factor receptor, *TKI* tyrosine kinase inhibitor.

A total of 185 patients were treated with first-line EGFR TKIs for advanced NSCLC at the study institution during the study period (Fig. 1). Among 185 patients, 31 had no EGFR gene mutation. Sixteen patients received first-line EGFR-TKIs within 30 days; one died 1 day after the initiation of EGFR-TKIs, and 2 patients were not followed up because they were transferred to another hospital, and the other 13 patients discontinued first-line EGFR-TKIs within 30 days due to adverse events (hepatic dysfunction, N = 5; gastrointestinal toxicity, N = 4; heart failure, N = 1; pancreatitis, N = 1; fever, N = 1; anemia, N = 1). Therefore, the remaining 138 patients were included in the final analyses. Among them, 32 patients orally received minocycline prophylactically or therapeutically for 30 days or longer during the administration of EGFR-TKIs as first-line therapy. Thirty-two patients were categorized into the MINO group, whereas the remaining 106 were categorized into the control group (Fig. 1).

The baseline characteristics of the study cohort at the initiation of first-line EGFR-TKIs are summarized in Table 1. No significant differences were observed in age, tissue type, or the type of EGFR gene mutation between the two groups. Different types of first-line EGFR-TKIs were administered to the two groups because the frequency of skin rash differed depending on the type of EGFR-TKI; more patients received erlotinib and afatinib in the MINO group (37.5 and 37.5%, respectively), whereas more received gefitinib and osimertinib in the control group (40.6 and 34.0%, respectively) (Table 1). None of the patients were administered a combined treatment of first-line EGFR-TKI and either a vascular endothelial growth factor (VEGF) inhibitor or a vascular endothelial growth factor receptor (VEGFR) inhibitor. In the MINO group, patients started minocycline at a median of 14.5 days (range 0–1133 days) after the initial administration of first-line EGFR-TKIs, and received minocycline for a median of 388.5 days (range 33–2396 days) (Table 1).

**Table 1:** Baseline characteristics of patients at the initiation of first-line EGFR-TKIs.

Variables	All (N = 138)	MINO + (N = 32)	MINO – (N = 106)	P value
Age, years	70 (23–88)	68 (39–85)	71 (23–88)	0.078
Male	36 (26.1)	10 (31.2)	26 (24.5)	0.49
Smoker	37 (26.8)	11 (35.5)	26 (24.8)	0.26
Adenocarcinoma	135 (97.8)	31 (96.9)	104 (98.1)	0.55
EGFR mutation				
Ex19 deletion	62 (44.9)	16 (50)	46 (43.4)	0.64
Ex21 L858R	62 (44.9)	12 (37.5)	50 (47.2)	
Minor mutation	14 (10.1)	4 (12.5)	10 (9.4)	
First-line EGFR-TKIs				
Gefitinib	49 (35.5)	6 (18.8)	43 (40.6)	< 0.001
Erlotinib	34 (24.6)	12 (37.5)	22 (20.8)	
Afatinib	17 (12.3)	12 (37.5)	5 (4.7)	
Osimertinib	38 (27.5)	2 (6.2)	36 (34.0)	
Combination of first-line EGFR-TKI and VEGF or VEGFR inhibitor	0 (0)	0 (0)	0 (0)	1.0
High expression of PD-L1	7 (5.1)	2 (6.2)	5 (4.7)	0.14
Recurrence after surgery	56 (40.6)	11 (34.4)	45 (42.5)	0.14
Days from 1st EGFR-TKI administration to minocycline administration, days			14.5 (0–1133)	
Duration of minocycline administration, days			388.5 (33–2396)	

Data are presented as medians (range) or N (%).

*MINO* minocycline, *EGFR* epidermal growth factor receptor, *TKI* tyrosine kinase inhibitor, *VEGF* vascular endothelial growth factor, *VEGFR* vascular endothelial growth factor receptor, *PD-L1* programmed death ligand 1.

### Adverse events of EGFR-TKIs

Skin rash occurred more often as an adverse event of EGFR-TKIs in the MINO group than in the control group (84.4% vs. 57.5%, p = 0.0062) (Table 2). Similarly, gastrointestinal toxicity occurred more often in the MINO group than in the control group (43.8% vs. 16.0%, p = 0.0029) (Table 2). One patient in the MINO group and 12 patients in the control group discontinued first-line EGFR-TKIs due to adverse events (hepatic dysfunction, N = 7; skin rash, N = 2; lung injury, N = 2; gastrointestinal toxicity, N = 1; heart failure, N = 1).

**Table 2:** Adverse events of first-line EGFR-TKIs.

Variables	All (N = 138)	MINO + (N = 32)	MINO – (N = 106)	P value
Skin rash	88 (63.8)	27 (84.4)	61 (57.5)	0.0062
Hepatic dysfunction	31 (22.5)	9 (28.1)	22 (20.8)	0.47
Gastrointestinal toxicity	31 (22.5)	14 (43.8)	17 (16.0)	0.0029
Renal dysfunction	3 (2.2)	1 (3.1)	2 (1.9)	1.0
Lung injury	2 (1.4)	0 (0)	2 (1.9)	1.0
Heart failure	2 (1.4)	1 (0)	1 (1.9)	0.41
Neutropenia	1 (0.72)	0 (0)	1 (1.9)	1.0
Neuropathy	1 (0.72)	0 (0)	1 (1.9)	1.0

Data are presented as N (%).

*MINO* minocycline, *EGFR* epidermal growth factor receptor, *TKI* tyrosine kinase inhibitor.

### Treatment efficacy of first-line EGFR-TKIs

**Figure 2:**
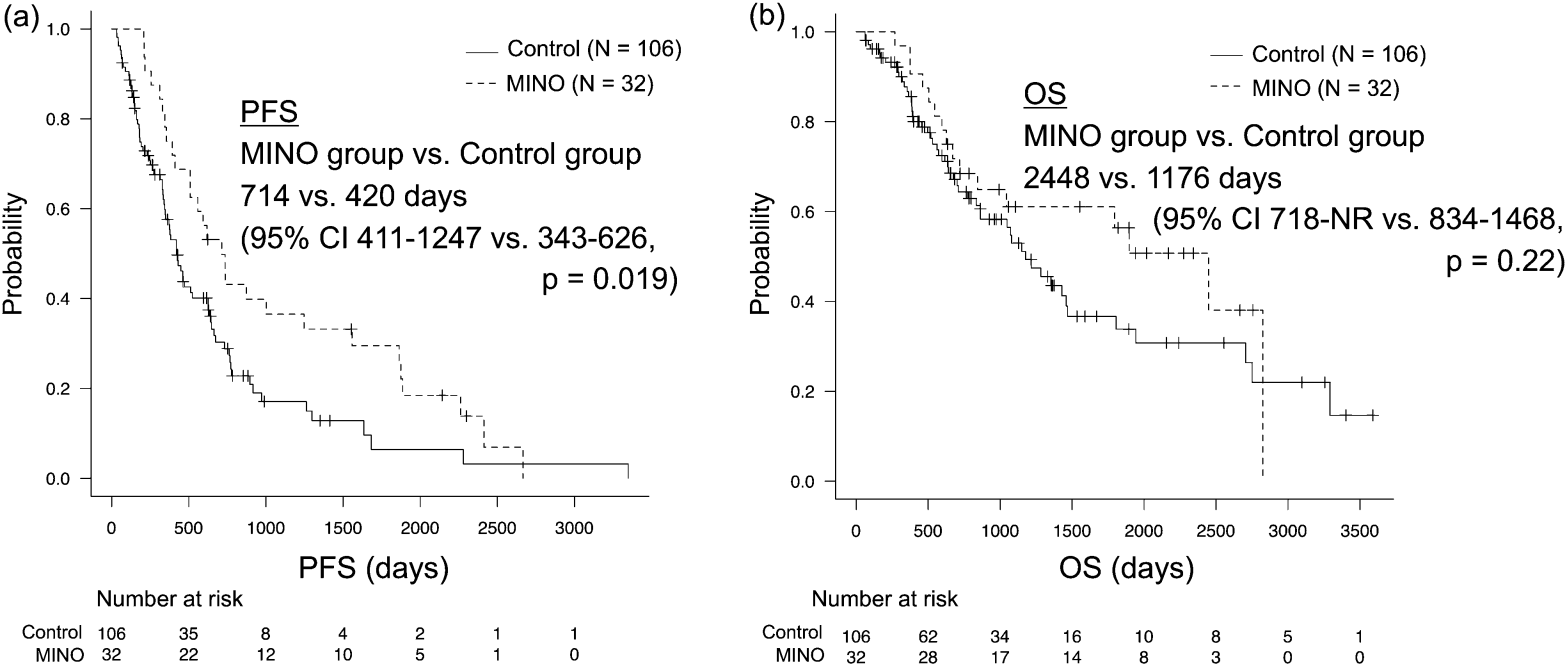
Kaplan–Meier curves of (**a**) progression-free-survival (PFS) and (**b**) overall survival (OS) with first-line epidermal growth factor receptor tyrosine kinase inhibitors (EGFR-TKIs) in two groups of patients who received minocycline (MINO) for skin rash as an adverse event of EGFR-TKIs (MINO group) and those who did not (control group). *NR* not reached.

The treatment efficacy of first-line EGFR-TKIs significantly differed between the two groups (MINO group vs. control group; CR, 10.0% vs. 1.1%; PR, 76.7% vs. 69.9%; SD, 13.3% vs. 26.9%; PD, 0% vs. 2.2%; p = 0.048) (Table 3, Supplementary Table S1). ORR to first-line EGFR-TKIs was slightly higher in the MINO group than in the control group (86.7% vs. 71.0%, p = 0.096). Median PFS with first-line EGFR-TKIs was significantly longer in the MINO group than in the control group; 714 (95% confidence interval CI 411–1247) days vs. 420 (95% CI 343–626) days, p = 0.019 (Fig. 2, Supplementary Table S2). Similarly, median OS with first-line EGFR-TKIs was slightly longer in the MINO group than in the control group; 2448 (95% CI 718–NR) days vs. 1176 (95% CI 834–1468) days, p = 0.22 (Fig. 2, Supplementary Table S2). On the other hand, median PFS and OS were not significantly longer in patients with skin rash as an adverse event of EGFR-TKIs than in those without skin rash (PFS 508 [95% CI 411–647] days vs. 382 [95% CI 328–731] days, p = 0.47; OS 1216 [95% CI 843–2825] days vs. 1428 [95% CI 776–2448] days, p = 0.68) (Supplementary Fig. S1).

**Table 3:** Treatment efficacy of first-line EGFR-TKIs.

	All (N = 123)*	MINO + (N = 30)	MINO – (N = 93)	P value
Treatment efficacy				
CR	4 (3.3)	3 (10.0)	1 (1.1)	0.048
PR	88 (71.5)	23 (76.7)	65 (69.9)	
SD	29 (23.6)	4 (13.3)	25 (26.9)	
PD	2 (1.6)	0 (0)	2 (2.2)	
ORR	74.8%	86.7%	71.0%	0.096

Data are presented as N (%).

*EGFR* epidermal growth factor receptor, *TKI* tyrosine kinase inhibitor, *MINO* minocycline, *CR* complete response, *PR* partial response, *SD* stable disease, *PD* progressive disease, *ORR* overall response rate.

*Eleven patients had no target lesion that was assessable based on the Response Evaluation Criteria in Solid Tumors version 1, and four patients did not undergo an imaging assessment.

Moreover, among patients without skin rash (N = 50), median PFS was significantly longer in patients taking minocycline (N = 5) than in those not taking minocycline (N = 45) (1886 [95% CI 216-NR] vs. 347 [95% CI 245–644], p = 0.027) (Supplementary Fig. S2). Similarly, median OS was slightly longer in patients taking minocycline than in those not taking minocycline among patients without skin rash (2448 [95% CI 1796-NR] vs. 1048 [95% CI 701–2705], p = 0.17) (Supplementary Fig. S2).

### Factors influencing the treatment efficacy of first-line EGFR-TKIs

The multivariate analysis confirmed that the administration of minocycline for 30 days or longer correlated with good PFS and OS with first-line EGFR-TKIs (PFS HR 0.44 [95% CI 0.27–0.73], p = 0.0014; OS HR 0.50 [95% CI 0.27–0.92], p = 0.027) (Table 4). Receiving first-line Osimertinib correlated with good PFS, and recurrence after surgery also correlated with good PFS and OS (Table 4, Supplementary Fig. S3). However, skin rash as an adverse event of EGFR-TKIs did not significantly influence the treatment efficacy of first-line EGFR-TKIs (PFS HR 0.86 [95% CI 0.54–1.35], p = 0.51; OS HR 0.73 [95% CI 0.40–1.33], p = 0.30).

**Table 4:** Multivariate analysis of factors influencing the treatment efficacy of first-line EGFR-TKIs.

Variables	HR	95% CI	P value
(a) Multivariate analysis of factors influencing PFS with first-line EGFR-TKIs			
Minocycline over 30 days	0.44	0.27–0.73	0.0014
Age > 75	0.89	0.56–1.44	0.64
Male	0.76	0.36–1.58	0.46
Smoking	1.60	0.77–3.36	0.21
EGFR minor mutation	0.81	0.41–1.62	0.56
Osimertinib	0.49	0.26–0.92	0.027
Recurrence after surgery	0.49	0.26–0.92	0.0010
Skin rash	0.86	0.54–1.35	0.51
Hepatic dysfunction	1.28	0.74–2.19	0.38
Gastrointestinal toxicity	1.25	0.76–2.06	0.38
(b) Multivariate analysis of factors influencing OS with first-line EGFR-TKIs			
Minocycline over 30 days	0.50	0.27–0.92	0.027
Age > 75	1.27	0.72–2.25	0.41
Male	0.55	0.23–1.30	0.17
Smoking	2.14	0.92–4.99	0.078
EGFR minor mutation	0.88	0.37–2.10	0.78
Osimertinib	0.38	0.13–1.12	0.079
Recurrence after surgery	0.48	0.27–0.83	0.0085
Skin rash	0.73	0.40–1.33	0.30
Hepatic dysfunction	0.74	0.38–1.44	0.37
Gastrointestinal toxicity	1.66	0.91–3.03	0.098

*EGFR* epidermal growth factor receptor, *TKI* tyrosine kinase inhibitor, *HR* hazard ratio.

## Discussion

In the present retrospective cohort study, we showed that the prophylactic or therapeutic administration of minocycline for skin rash prolonged PFS in EGFR-mutant NSCLC patients treated with EGFR-TKIs. Moreover, the administration of minocycline was identified as an independent prognostic factor for PFS and OS.

Although previous studies reported that skin rash induced by EGFR-TKIs was associated with better outcomes, the results obtained herein indicated that it was not a prognostic factor. In the present study, the administration of osimertinib, but not other TKIs, was an independent prognostic factor for PFS. This result is consistent with the findings of the FLAURA trial, which indicated that osimertinib showed superior treatment efficacy to other TKIs as the first-line treatment for EGFR-mutant advanced NSCLC^9,10^. Previous findings demonstrated that the frequency of skin rash induced by osimertinib was less than that with other EGFR-TKIs^9,10^. In the present study, only 2 out of 38 patients receiving osimertinib took minocycline for skin rash. Therefore, we estimated that skin rash was not a prognostic factor in this study because of differences in treatment efficacy and the frequency of skin rash between osimertinib and other TKIs.

The results obtained herein indicated that the administration of minocycline was an independent prognostic factor for PFS and OS. In the present study, the administration of minocycline was limited to NSCLC patients with EGFR mutations treated with EGFR-TKIs. Since minocycline was administered for skin rash induced by EGFR-TKIs, the timing and duration of minocycline administration depended on adverse events and the treatment duration of EGFR-TKIs. Therefore, the OS of patients treated with minocycline was restricted by the administration of EGFR-TKIs. To overcome this limitation, a prospective study to validate the treatment efficacy of minocycline for NSCLC patients with and without EGFR mutations is needed.

In the present study, the administration of minocycline prolonged the PFS of EGFR-mutant NSCLC patients independently of skin rash. However, the mechanism of action of minocycline for improvements in the outcomes of EGFR-mutant NSCLC patients remain unclear. Minocycline has been reported to have various chemical properties^22–27^. Previous studies reported the effects of minocycline on non-bacterial infections (virus, protozoa, and helminth)^22,23^, rheumatoid arthritis^24^, neurological disease^25^, and cancer^26,27^. We also showed that minocycline enhanced antitumor T cell responses^28^. Currently, we have conducted a clinical study on the T cell responses of COVID-19 patients treated with tetracyclines (trial registration number: jRCTs051200049). We are in the process of identifying the molecular target of tetracyclines in this mechanism of action, which has the potential to be applied to cancer immunotherapy. A previous study has indicated that EGFR-mutant NSCLC is characterized by a high infiltration of CD4 + effector regulatory T cells, which can be reduced by the administration of EGFR inhibitors in in vivo experiments. Additionally, the combination of EGFR inhibitors with anti–PD-1 immunotherapy exhibits superior in vivo antitumor effects when compared to either treatment alone^29^. Further studies to elucidate the mechanisms of action of minocycline will contribute to the development of novel therapeutics for lung cancer.

The present study has several limitations. This was a retrospective study performed at a single institution, including a heterogenous cohort of patients treated with several types of EGFR-TKIs. Furthermore, we did not investigate the relationship between the severity of skin rash and the treatment efficacy of EGFR-TKIs because we did not obtain detailed information on the grade of skin rash, namely, the extent to which skin rash covered the body surface area of patients. A large-scale prospective cohort study is needed to investigate the relationship between the administration of minocycline and prognosis of lung cancer patients.

In conclusion, the administration of minocycline was identified as a factor that positively contributed to the treatment efficacy of first-line EGFR-TKIs independently of skin rash. The present results suggest that minocycline improves the prognosis of lung cancer patients based on an unknown mechanism.

## Methods

### Patient selection and data collection

This retrospective cohort study included patients with stage IV, unresectable stage III, or postoperative recurrent EGFR-mutant NSCLC treated with EGFR-TKIs at Osaka University Hospital between January 2010 and June 2021. Data were collected from medical charts. We collected data on baseline characteristics, the treatment efficacy and adverse events of EGFR-TKIs, and the prognosis of patients. Among patients treated with EGFR-TKIs more than once, data related to the administration of EGFR-TKIs as first-line therapy were included in the present study. Patients with lung cancer harboring no EGFR mutations or administered first-line EGFR-TKIs for less than 30 days were excluded.

The present study mainly focused on patients who orally received minocycline prophylactically or therapeutically for skin rash as an adverse event of EGFR-TKIs. Patients who received minocycline for 30 days or longer during the administration of EGFR-TKIs as first-line therapy were grouped into the ‘MINO group’, and the remaining patients were grouped into the ‘control group’. Attending physicians decided whether to administer minocycline prophylactically or therapeutically to patients for skin rash. We compared the treatment efficacy of EGFR-TKIs between the MINO and control groups.

The therapeutic effectiveness of EGFR-TKIs was appraised by gauging the overall response rate (ORR), progression-free survival (PFS), and overall survival (OS). The attending physician evaluated the response to EGFR-TKIs based on the Response Evaluation Criteria in Solid Tumors version 1.1 (RECIST 1.1). The duration of PFS was defined as the period between the initiation of EGFR-TKIs and the point of disease progression. The duration of OS was defined as the time from the initiation of EGFR-TKIs to the time of death from any cause. The adverse events that occurred during the period of observation were assessed based on the National Cancer Institute Common Terminology Criteria for Adverse Events (Version 5.0).

### Statistical analysis

To assess the factors correlated with the administration of minocycline, we employed Fisher's exact test for categorical data and the Mann–Whitney *U* test for numerical data. The chi-squared test was used to compare the objective response rate (ORR) of each treatment. We generated progression-free survival (PFS) and overall survival (OS) curves using the Kaplan–Meier method and compared them using the Log-rank test. Additionally, a multivariate analysis of PFS and OS was conducted using Cox proportional hazard regression. Descriptive statistics, including medians, frequencies, and percentages, were reported in this study. All p-values reported were two-tailed, and those below 0.05 were deemed statistically significant. We performed all statistical analyses using EZR (Saitama Medical Center, Jichi Medical University, Saitama, Japan), a graphical user interface for R (the R Foundation for Statistical Computing, Vienna, Austria).

### Ethical considerations

This retrospective study was approved by the Institutional Review Board of Osaka University Hospital (No. 22097). Due to the retrospective study design and based on the Japanese ethical guidelines for clinical research, the requirement for informed consent was waived. The waiver of informed consent was approved by the Institutional Review Board of Osaka University Hospital. The present study was conducted according to the principles of the Declaration of Helsinki.

## Supplementary Information

Supplementary Information.

## Data Availability

The datasets generated during the current study are available from the corresponding author on reasonable request.
