# Supplementary material for: Impact of minocycline on outcomes of EGFR-mutant non-small cell lung cancer patients treated with EGFR-TKIs
Source: Sci Rep. 2023 May 23;13:8313. doi: 10.1038/s41598-023-35519-4 (PMC10204024; doi:10.1038/s41598-023-35519-4)

Impact of minocycline on outcomes of EGFR-mutant non-small cell lung cancer patients treated with EGFR-TKIs

Mari Tone[1], Kota Iwahori[1,2]*, Takayuki Shiroyama[1], Shinji Futami[1], Yujiro Naito[1], Kiyoharu Fukushima[1], Kotaro Miyake[1], Shohei Koyama[1], Haruhiko Hirata[1], Izumi Nagatomo[1], Hisashi Wada[2], Yoshito Takeda[1], Atsushi Kumanogoh[1,3,4,5,6,7]

1 Department of Respiratory Medicine and Clinical Immunology, Osaka University Graduate School of Medicine, Osaka, Japan

2 Department of Clinical Research in Tumor Immunology, Osaka University Graduate School of Medicine, Osaka, Japan

3 Department of Immunopathology, World Premier International Research Center Initiative (WPI), Immunology Frontier Research Center (IFReC), Osaka University, Osaka, Japan.

4 Integrated Frontier Research for Medical Science Division, Institute for Open and Transdisciplinary Research Initiatives (OTRI), Osaka University, Osaka, Japan.

5 Center for Infectious Diseases for Education and Research (CiDER), Osaka University, Osaka, Japan.

6 Japan Agency for Medical Research and Development – Core Research for Evolutional Science and Technology (AMED–CREST), Osaka University, Osaka, Japan

7 Center for Advanced Modalities and DDS (CAMaD), Osaka University, Osaka, Japan

*To whom correspondence should be addressed:

Kota Iwahori

Department of Clinical Research in Tumor Immunology, Graduate School of Medicine, Osaka University, 2-2 Yamadaoka, Suita, Osaka 565-0871, Japan.

E-mail address: iwahori@climm.med.osaka-u.ac.jp

Supplementary Table S1. Treatment efficacy of first-line EGFR-TKIs

	ORR	PFS, days	OS, days
Gefitinib<br>(N = 49)	69.8%	419<br>(95% CI 328-647)	1176<br>(95% CI 701-2448)
Erlotinib<br>(N = 34)	70.0%	349<br>(95% CI 211-460)	863<br>(95% CI 544-1353)
Afatinib<br>(N = 17)	100%	736<br>(95% CI 411-1635)	NR<br>(95% CI 626-NR)
Osimertinib<br>(N = 38)	72.7%	735<br>(95% CI 429-NR)	NR<br>(95% CI NR-NR)

EGFR, epidermal growth factor receptor; TKI, tyrosine kinase inhibitor; ORR, overall response rate; PFS, progression-free survival; OS, overall survival; NR, not reached.

Supplementary Table S2. Treatment efficacy of EGFR-TKIs in 14 patients who received minocycline within 30 days in the control group

	Minocycline within 30 days in the control group
Duration of minocycline administration, days	11.5 (1-25) *
ORR	64.3%
PFS, days	328 (95% CI 168-896)
OS, days	776 (95% CI 364-1806)

*Data are presented as medians (range).
EGFR, epidermal growth factor receptor; TKI, tyrosine kinase inhibitor; ORR, overall response rate; PFS, progression-free survival; OS, overall survival; NR, not reached.

Supplementary Figure S1. Kaplan–Meier curves of (a) progression-free-survival (PFS) and (b) overall survival (OS) with first-line epidermal growth factor receptor tyrosine kinase inhibitors (EGFR-TKIs) in two groups of patients who developed skin rash as an adverse event of EGFR-TKIs and those who did not.

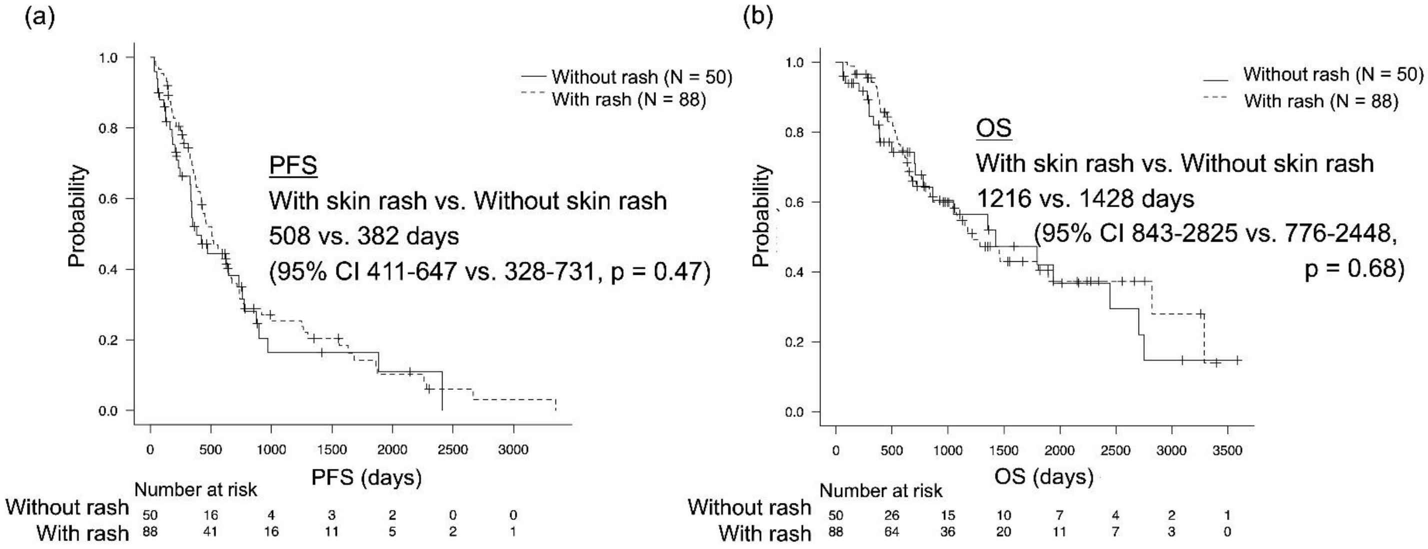

Supplementary Figure S2. Kaplan–Meier curves of (a) progression-free-survival (PFS) and (b) overall survival (OS) with first-line epidermal growth factor receptor tyrosine kinase inhibitors (EGFR-TKIs) in two groups among patients who did not develop skin rash. The two groups consisted of patients who prophylactically received minocycline (MINO) for skin rash as an adverse event of EGFR-TKIs (MINO groups) and those who did not (control group). NR, not reached.

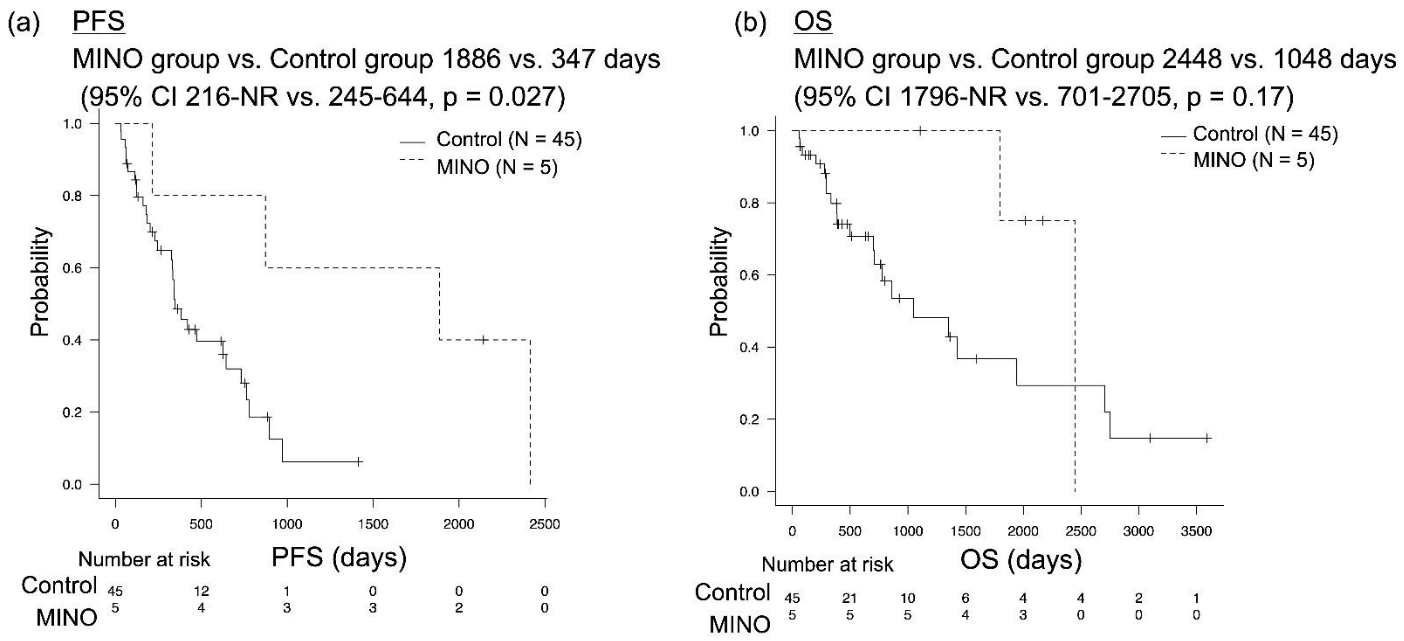

Supplementary Figure S3. Kaplan–Meier curves of (a) progression-free-survival (PFS) and (b) overall survival (OS) with each first-line epidermal growth factor receptor tyrosine kinase inhibitor (EGFR-TKI) in all patients. NR, not reached.

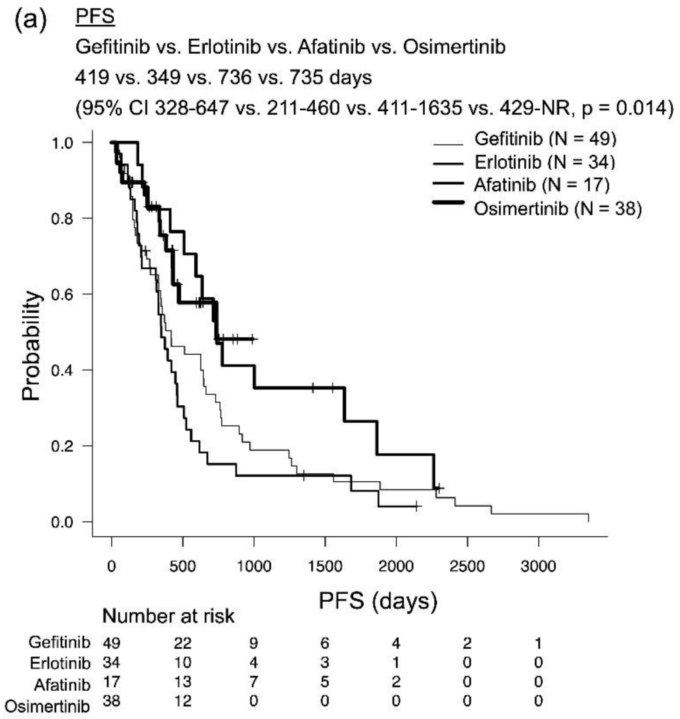

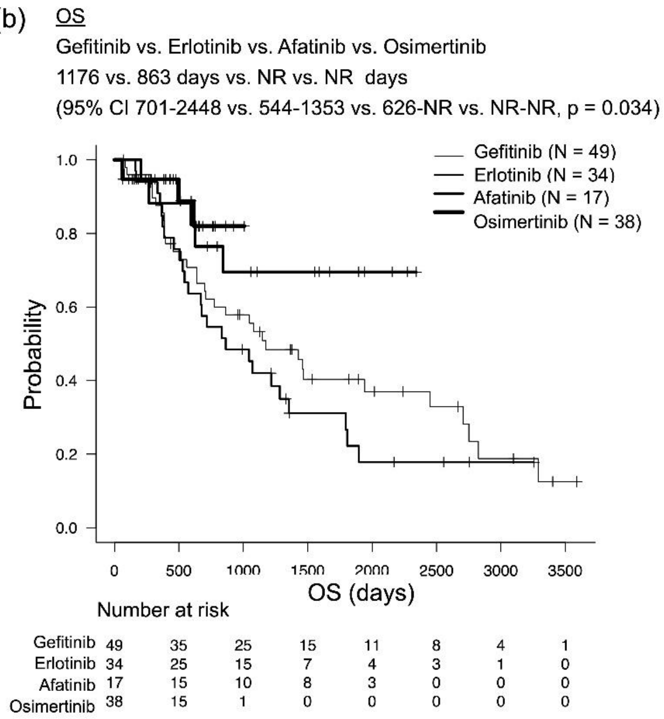

Supplement: Supplementary file 1 — Supplementary Information. [file 41598_2023_35519_MOESM1_ESM.pdf]
